# Gynaecological Society of Boston

**Published:** 1870-08

**Authors:** 


*Gynaecological Society of Boston*:

We have received the By-laws, Constitution List of Officers. etc., etc., of this society. It is plain to see, by its monthly journal, its valuable contributions and reports, and by its general activity and prosperity that this organization is, and is to become a great power, situated at the "hub," which will make its influence felt for progress and reform.

